# Case of Chorea, Cured by the External Application of Tartar Emetic, after Purging and Tonics Had Failed

**Published:** 1826-10

**Authors:** Æneas M'Andrew

**Affiliations:** Physician to the South London Dispensary.


### CHOREA.

*Case of Chorea, cured by the external Application of Tartar Emetic, after Purging and Tonics had failed.* By ÆNEAS M'ANDREW, M.D. Physician to the SOUTH LONDON DISPENSARY.

MARIA CHARLES, aged nine, was admitted a patient at the South London Dispensary on the 9th March, 1826. Her complaints began in January, at which time she was observed to be labouring under slight convulsive motions of both sides of the body. These were always increased by mental agitation, and have never left her since that time. She also complained of occasional pain in the head, and was affected with a diarrhœa, which lasted nearly three weeks. At school, the convulsive movements were attributed to a bad habit, and she was punished accordingly. Her brother has for some years been subject to epileptic fits, and his intellect is now much weakened. Her mother labours under constant trembling of the head.

When examined to-day, the motions were found to be confined more particularly to the extremities of the right side; she walks unsteadily, but can guide a glass to her mouth easily enough. Pulse weak; appetite good; bowels rather costive.

> ℞. Hydrar. Submuriat. gr. iij.; Pulv. Jalapæ gr. xv. M. fiat pulvis omni nocte sumendus.—She afterwards took an increased dose: ℞. Hydr. Subm. gr. v.; Pulv. Jalapæ ℈j. M.

April 10th.—She has taken the powders regularly up to this period, and has had about three alvine evacuations daily; they at first were dark-coloured and fetid, but are now quite natural in their appearance. Large doses of medicine were required to open her bowels. Leeches were at one time applied to relieve the pain of the head. She became much thinner; and the convulsions, although at times apparently slighter, were, upon the whole, rather more severe than at first.

> Sulphate of Zinc, Extract of Gentian, and the shower-bath, were latterly conjoined with the purgatives; but the two first were stopped, in consequence of producing vomiting.

April 17th.—Has taken, since the 13th, some Castor-oil, to keep her bowels regularly open; and a draught, composed of Sulphuric Æther, Tincture of Valerian, and Tincture of Opium, was ordered to be taken every night. Is now decidedly worse; the convulsive movements are incessant; they affect both sides of the body, and are said to be worse during sleep. She can scarcely stand or walk, and it is now impossible for her to carry a glass to her mouth.

When desired to put out her tongue, she makes a great many awkward attempts, but cannot succeed. Speech is much affected. The motions were formerly diminished on the approach of a stranger, they are now increased by that circumstance. Bowels open once a-day; appetite good.

The draught was immediately suspended, and the purging powder repeated.

20th.—Continues much the same.

The head was ordered to be shaved, and a little of the Tartar-emetic Ointment to be rubbed on the scalp night and morning.—The powders to be continued.

24th.—The ointment has produced a copious eruption of pustules over the upper part of the head, and a few at the lower part: they only appeared yesterday. Convulsive motions of the right side considerably diminished, but on the left side they are still violent. She now articulates distinctly, and can put out her tongue, but quickly draws it in again. Being desired to carry a glass of water to her mouth, she made the attempt, but could not succeed: her face got flushed, she seemed irritated, and the motions became very strong. Bowels open about three times a-day; appetite keen.

The same plan to be continued.

27th.—Continues to improve. Convulsive motions less severe, and at times almost gone, or confined to the lower limbs. She articulates distinctly, but cannot advance even a few steps without being supported. Motions still persist during the night-time; bowels freely opened; appetite keen. The whole of the head covered with pustules.

The ointment to be rubbed into the upper part of the spine.

May 1st.—Her mother states that for two days she was unable to articulate. Motions less. When told to remain still, she can do so for a short time. She attempted to walk, but tottered very much, and stumbled. Articulation distinct. Eruption beginning to appear on the back part of the neck.—Continue.

11th.—Continued to improve gradually for two days, and then remained stationary till the 8th, since which time she has been very much better. She can now walk without any assistance, although her steps are still irregular. Motions are very slight, and cease entirely when she is desired to remain quiet. She sleeps well, and almost without any irregular motion. Eruption has been maintained on the head and upper part of the spine. There appeared yesterday a swelling on the forehead, extending from the hairy scalp to the root of the nose: it is painful, but colourless, nor is the pain increased on pressure. She feels thirsty; appetite good; pulse natural. Has gained flesh.

The friction to be stopped, and a lotion of the Subacetate of Lead to be applied to the swelling. The powders to be continued.

13th.—The swelling yesterday left the forehead, and passed to the eyelids and roots of the nose, where it now remains, but considerably diminished in size. She walked about a mile and a half to come to the Dispensary. For about an hour after rising from bed, she is affected with the movements, which are not severe, and leave her almost entirely during the rest of the day. Eruption drying.

Continue.

29th.—Has continued to improve steadily. Motions are now very trifling, and confined to the fingers of the left hand, for a short time in the morning. She can work with her needle. The eruption is nearly healed, and some weakness, which remained in the left side of the body, is almost gone. Appetite good; bowels open, a dose of calomel and jalap being given occasionally. She took six powders with her, to be administered as her bowels might require it, and was dismissed from the Institution. I have seen her since, and assured myself that the recovery is complete.

The foregoing case was certainly not very severe when it first came under treatment, although the existence of convulsive affections in other members of the family indicated a predisposition to affections of that class, which rendered the prognosis very unfavourable. The previous diarrhœa, and the actual confined state of the bowels, naturally led to the opinion that some irritation of the intestinal canal was the exciting cause of the complaint; and accordingly the treatment recommended by Dr. HAMILTON was adopted to its full extent, and persevered in for more than a month. Large doses of purgative medicine were required to open the bowels freely; and the fæces, at first of an unhealthy aspect, gradually assumed their natural colour. This, however, was the only symptom of amendment; the convulsive motions were rather increased than diminished, and the emaciation advanced rapidly. Tonics having been rejected by the stomach, I resolved to try if a full dose of the more powerful antispasmodics would have any effect in arresting the motions. This, however, only did harm: she became much worse in consequence; every symptom was aggravated; and, although her appetite remained good, the consequences that might result from the convulsions persisting in her debilitated state, became the subject of serious apprehension. I was, therefore, induced to try the friction of the scalp by the tartar-emetic ointment; a plan of treatment, for which we are indebted to Mr. HUNTER, of Glasgow, who has published a very decided instance of success arising from it in the Edinburgh Medical and Surgical Journal, for April 1825. I believe that the case of Maria Charles is another example of its efficacy: a decided improvement followed the eruption, and, under its influence, the motions of the body were at last brought back to their natural state. It is, however, a severe remedy, and in this case produced a swelling that might have terminated in erysipelas; and I apprehend that it should not be had recourse to until purging has been fairly tried; which, when succeeded by tonics, is, in the majority of cases, sufficient to effect a cure.

Besides, the adoption of this plan does not depend upon empiricism merely: we can, in some degree, explain the manner in which it proves beneficial. We know that a morbid accumulation of fæces is the most common cause of the complaint, and that the most important indication to be fulfilled is its removal. The explanation of the good effects that have sometimes resulted from other plans, rests upon hypothesis merely: no other exciting cause has been sufficiently established, and we have not as yet derived any assistance from pathological anatomy. We are, therefore, deprived of a basis on which to found our treatment. We recommend it because it did good before; but, being ignorant of the particular case to which it is applicable, we cannot be surprised if it fails, and cannot feel perfect satisfaction if it succeeds.

*Great Surrey-street*; *July* 1826.

---

